# Xuefu zhuyu decoction improves cognitive impairment in experimental traumatic brain injury via synaptic regulation

**DOI:** 10.18632/oncotarget.18895

**Published:** 2017-06-30

**Authors:** Jing Zhou, Tao Liu, Hanjin Cui, Rong Fan, Chunhu Zhang, Weijun Peng, Ali Yang, Lin Zhu, Yang Wang, Tao Tang

**Affiliations:** ^1^ Laboratory of Ethnopharmacology, Institute of Integrated Traditional Chinese and Western Medicine, Xiangya Hospital, Central South University, 410008 Changsha, China; ^2^ Department of Gerontology, Traditional Chinese Medicine Hospital Affiliate to Xinjiang Medical University, 830000 Urumqi, China; ^3^ Department of Traditional Chinese Medicine, 2nd Xiangya Hospital, Central South University, 410011 Changsha, China; ^4^ Department of Neurology, Henan Province People’ Hospital, 450003 Zhengzhou, China

**Keywords:** xuefu zhuyu decoction, traditional Chinese medicine, traumatic brain injury, cognitive impairment, synaptic regulation

## Abstract

An overarching consequence of traumatic brain injury (TBI) is the cognitive impairment. It may hinder individual performance of daily tasks and determine people's subjective well-being. The damage to synaptic plasticity, one of the key mechanisms of cognitive dysfunction, becomes the potential therapeutic strategy of TBI. In this study, we aimed to investigate whether Xuefu Zhuyu Decoction (XFZYD), a traditional Chinese medicine, provided a synaptic regulation to improve cognitive disorder following TBI. Morris water maze and modified neurological severity scores were performed to assess the neurological and cognitive abilities. The PubChem Compound IDs of the major compounds of XFZYD were submitted into BATMAN-TCM, an online bioinformatics analysis tool, to predict the druggable targets related to synaptic function. Furthermore, we validated the prediction through immunohistochemical, RT-PCR and western blot analyses. We found that XFZYD enhanced neuroprotection, simultaneously improved learning and memory performances in controlled cortical impact rats. Bioinformatics analysis revealed that the improvements of XFZYD implied the Long-term potentiation relative proteins including NMDAR1, CaMKII and GAP-43. The further confirmation of molecular biological studies confirmed that XFZYD upregulated the mRNA and protein levels of NMDAR1, CaMKII and GAP-43. Pharmacological synaptic regulation of XFZYD could provide a novel therapeutic strategy for cognitive impairment following TBI.

## INTRODUCTION

Traumatic brain injury (TBI) becomes an enormous socioeconomic burden worldwide [1, 2]. It is the leading cause of disability in the under 40 s [3]. Following TBI, cognitive problem is the key factor in determining people's subjective well-being and reducing quality of life in survivors [4]. Approximately 65% of patients with moderate to severe TBI, as well as 15% with mild TBI, have persistent cognitive deficit [5, 6]. Despite of substantial efforts, few satisfied therapeutic options are available to alleviate cognitive dysfunction after TBI in clinic [7, 8]. The reason is that the cognitive impairment with TBI involves complicated pathological mechanisms, thus “one-compound, one-target” based modern drugs are not available [9]. TBI can trigger cellular dysfunction/loss, neurotransmission impairments, synaptic connections disruption occur, and ultimately lead to the cognitive dysfunction [10–13]. Therefore, neuroscientists hope to identify new therapies for improvement of cognitive deficit after TBI.

Xuefu Zhuyu Decoction (XFZYD), a multi-components and well-known traditional Chinese medicine (TCM), was first described in the ancient Chinese document “Yilin Gaicuo” by Qingren Wang in the late Qing Dynasty. XFZYD consists of 11 crude herbs (The herbs and their component ratios are listed in Table 1). XFZYD has been widely used to treat the diseases that are caused by Qi stagnation and blood stasis according to TCM theory [14, 15]. XFZYD-derived compounds, such as hydroxysafflor yellow A, ferulic acid and gallic acid, were reported to protect against brain diseases [16–18]. Clinical and animal studies have confirmed the valid effects of XFZYD on cognitive disorder following TBI [19, 20]. However, the details concerning the pharmacological synaptic regulation of XFZYD on cognitive improvement after TBI remain unknown.

**Table 1:** Herbs and their component ratios of the xuefu zhuyu decoction

English name	Chinese name	Part used	ratio
Prunus persica (L.) Batsch	Taoren	Seed	8
Carthamus tinctorius L	Honghua	Flower	6
Ligusticumi chuanxiong Hort	Chuanxiong	Rhizome	3
Paeonia lactiflora Pall	Chishao	Root	4
Achyranthes bidentata Bl	Niuxi	Root	6
Rehmannia glutinosa Libosch	Dihuang	Root	6
Citrus aurantium L	Zhiqiao	Fruit	4
Bupleurum chinense DC	Chaihu	Root	2
Platycodon grandiflorum (Jacq.) A. DC	Jiegeng	Root	3
Angelicae sinensis (Oliv.) Diels	Danggui	Root	6
Glycyrrhiza uralensis Fisch	Gancao	Root and rhizome	4

The damage of synaptic plasticity mainly contributes to the cognitive deficit after TBI. TBI-mediated injuries may alter the expressions of neurotrophins, neurotransmitters and their receptors. Moreover, the expressions of synaptic structure proteins are also influenced following TBI. These changes in turn affect the excitatory postsynaptic potential, and contribute to cognitive function [21–23]. As the main form of synaptic plasticity, Long-term potentiation (LTP) is well recognized as the cellular mechanisms underlying learning and memory [24, 25]. The maintenance processes of LTP involve the N-methyl-D-aspartate receptor (NMDAR) opening, the Ca^2+^ influx, the Ca^2+^ binding to calmodulin, and the calcium calmodulin-dependent protein kinase II (CaMKII) rapid activation. The above cascades lead to the change of synaptic growth-associated proteins. The NMDAR controls the occurrence of LTP at individual synapses [26, 27]. Growth-associated protein-43 (GAP-43) is a marker of neuronal sprouting. GAP-43 is highly expressed in the brains of controlled cortical impact (CCI) rats with ongoing structural plasticity [28]. It highly enriched in axons to promote F-actin polymerization, leading to the activation of growth cone formation and regeneration following TBI [29]. CCI rat model reliably impairs LTP of synaptic efficacy [30]. The impairment of LTP can be persistent for a long time [31]. To active LTP induction may improve TBI-induced cognitive deficit [32]. Thus, synaptic regulation becomes the druggable strategy of cognitive rehabilitation after TBI.

Although the XFZYD neuroprotection to relieve the cognitive impairment following TBI is observed, underlying pharmacological mechanisms remain unclear. As far as we know, TCM implies the sophisticated multi-target effects because of its complicated compounds [33]. It is requisite to elucidate the multi-therapeutic mechanisms of XFZYD to alleviate the cognitive function of TBI. Fortunately, with the development of bioinformatics technology, increasing bioinformatic analysis websites are applied to reveal the multiple pharmacological effects of TCM [34–36]. In this study, BATMAN-TCM (available at http://bionet.ncpsb.org/batman-tcm), an online bioinformatics analysis tool for molecular mechanism of TCM, was used to profile the interaction among the key compounds of XFZYD, anchored proteins and pathways [37]. BATMAN-TCM renders the visualization of ingredient-target-pathway/disease association network and KEGG biological pathway. BATMAN-TCM is more comprehensive than other existed databases (e.g. TCM-ID, TCMSP, HIT) [37–40]. The bioinformatics analysis tends to help to understand the therapeutic mechanisms of XFZYD in treating TBI (“multi-components, multi-targets and multi-pathways”).

In the present study, we aimed to investigate whether XFZYD provided a synaptic regulation to improve cognitive function after TBI. The PubChem Compound IDs (CIDs) of the key compounds of XFZYD were submitted into BATMAN-TCM to predict the druggable targets related to synaptic function [37, 41]. We further verified this prediction by molecular biological methods (Immunohistochemical, RT-PCR and western blot analyses). The results may provide a novel therapeutic strategy of TCM to improve cognitive impairment after TBI.

## RESULTS

### XFZYD improved CCI-Induced neurologic deficit

**Figure 1:**
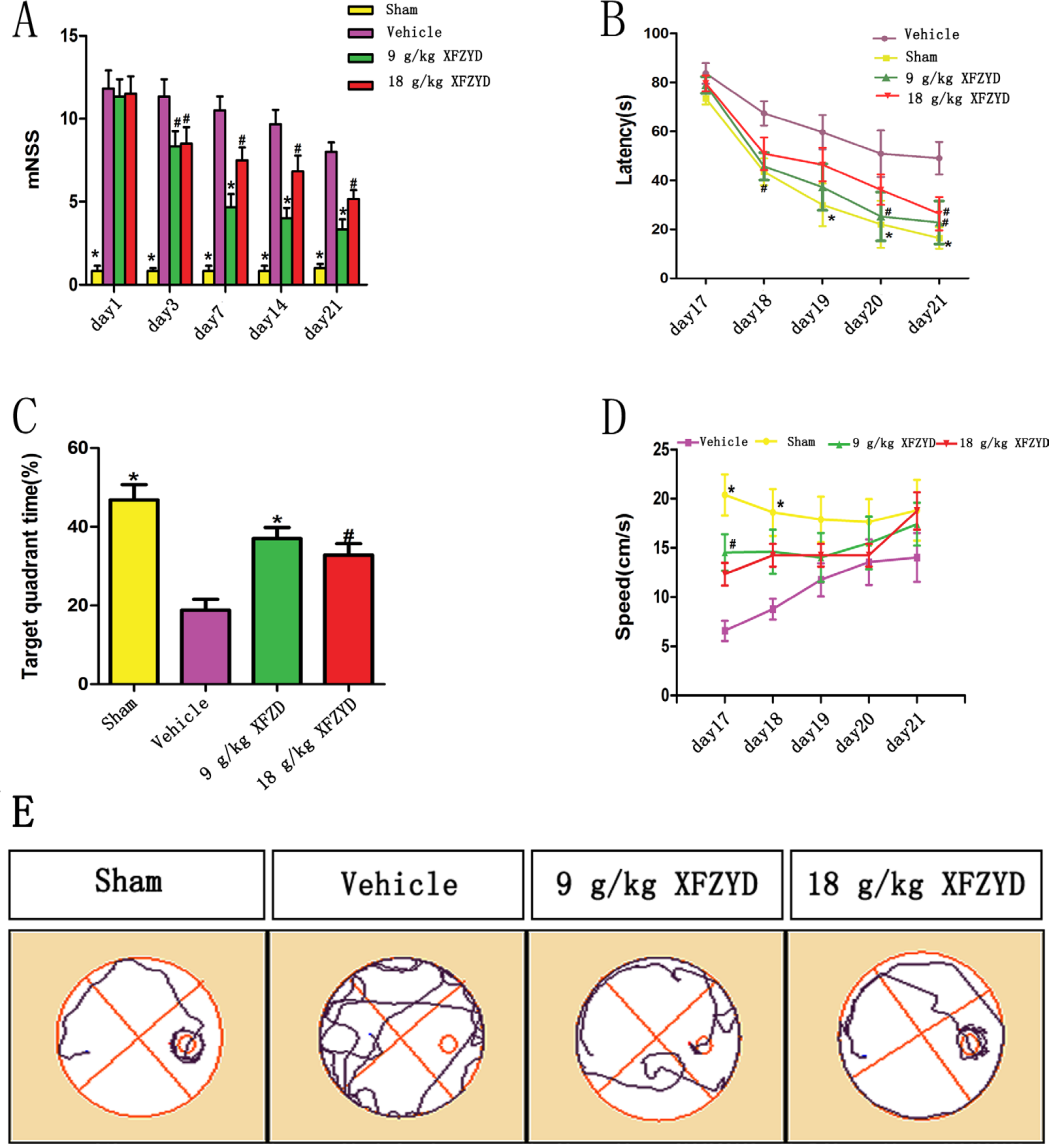
XFZYD improved neurological recovery, simultaneously enhanced spatial learning and memory acquisition after TBI (**A**) Rats exhibited neurologic deficit from the 1st to 21st day after TBI. The maximum neurologic deficit score was observed on the 1st day. Treatment with XFZYD (9 g/kg and 18 g/kg) significantly lowered the mNSS on the 3rd to 21st day compared with the Vehicle group. (**B**) Escape latency is the time that rats swim to reach the target platform. The escape latency was significantly longer in the Vehicle group than the Sham group from the 18th to the 21^st^ day. 9 g/kg of XFZYD significantly reduced the time on the 20th and 21st day compared with the Vehicle group, while 18 g/kg of XFZYD on the 21st day. (**C**) Compared with the Vehicle group, XFZYD (9 g/kg and 18 g/kg) increased the staying time in target quadrant in probe trial. (**D**) The swimming speed was impaired in CCI rats on the 17th and 18th day, the above impairment was reversed by XFZYD (9 g/kg) treatment on the 17th day. The swimming speed did not differ significantly between different groups on the 19th to 21st day. (**E**) Representative images of the swim paths on the 21st day after TBI. Rats treated with XFZYD were able to find the hidden platform more easily than rats with Vehicle. The values are expressed as the Mean ± SD, *n* = 8/group, *p < 0.01 and ^#^*p* < 0.05 vs. the Vehicle group.

CCI rats exhibited neurologic deficit on the 1st, 3rd, 7th, 14th, and 21st day (Figure 1A). On the 1st day, all the rats subjected to CCI showed similar neurological deficit, while the Sham group was almost free of neurological impairment. XFZYD (9 g/kg and 18 g/kg) improved neurological recovery on the 3rd, 7th, 14th and 21st day, as demonstrated by a decrease in modified neurological severity scores (mNSS) compared with the Vehicle group.

### XFZYD ameliorated CCI-Induced cognitive impairment

Morris water maze (MWM) test was performed among the four groups. CCI rats exhibited cognitive impairment in the MWM. The escape latency to find the hidden platform was longer in the Vehicle group than in the Sham group on the 18th to 21st day (Figure 1B, 1E). XFZYD (9 g/kg) significantly reduced the time on the 20th and 21st day compared with the Vehicle group, while XFZYD (18 g/kg) on the 21st day (Figure 1B). Percentage of time staying in the target quadrant in the probe trial was significantly shorter after CCI (Figure 1C), XFZYD (9 g/kg and 18 g/kg) treatment significantly increased the percentage on the 21st day compared with the Vehicle group (Figure 1C). The swimming speed was impaired in rats on the 17th and 18th day after CCI, but was increased by XFZYD (9 g/kg) treatment on the 17th day (Figure 1D).

### LTP was enriched by BATMAN-TCM combined with KEGG analysis

**Figure 2:**
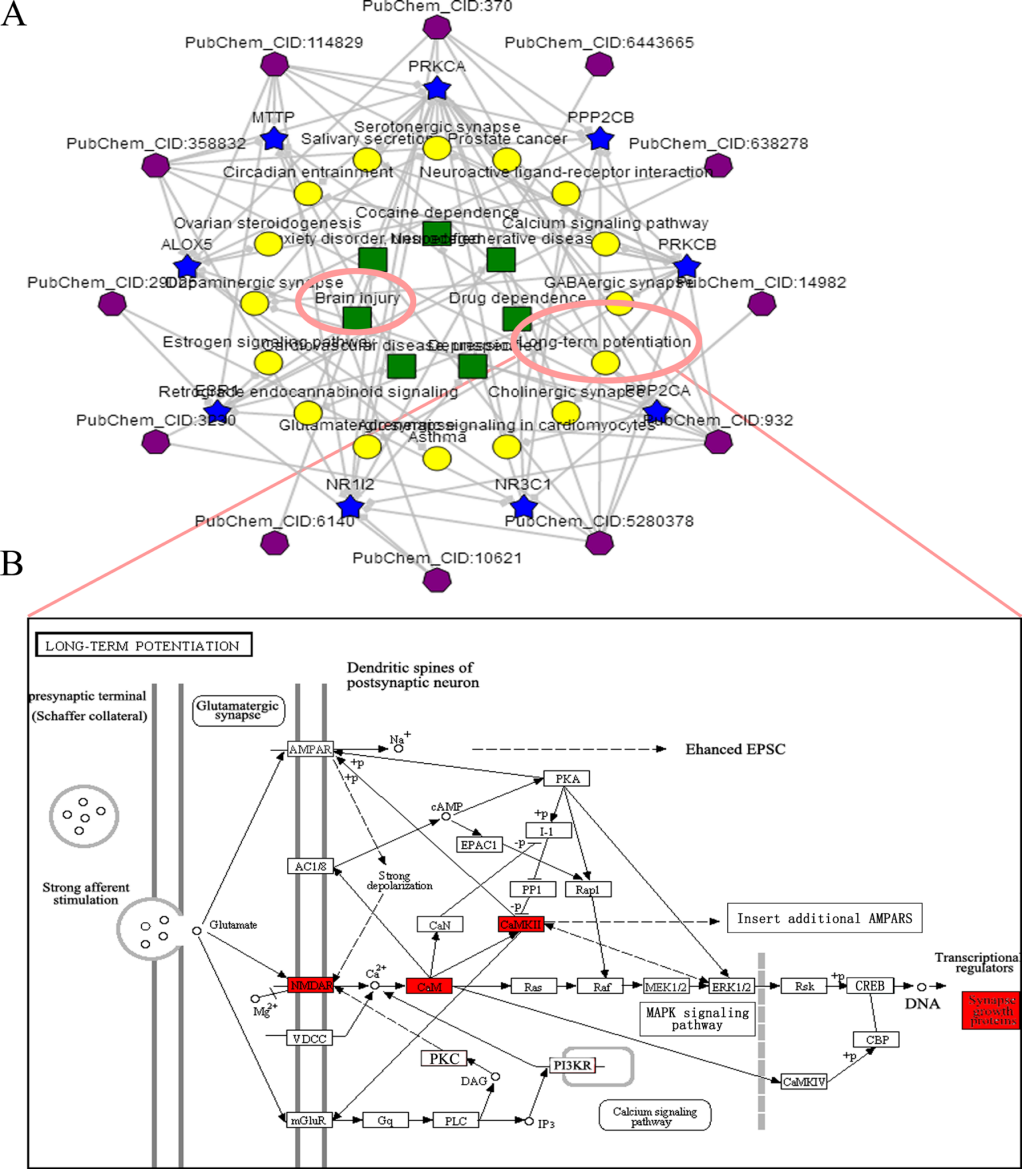
The ingredient-target-pathway analysis (**A**) Significantly enriched ingredient-target-pathway/disease association network was shown by an online bioinformatics analysis tool-BATMAN-TCM (http://bionet.ncpsb.org/batman-tcm). In the association network, there are four kinds of nodes including TCM's ingredients (sexangle, purple), targets (five-pointed star, blue), biological pathways (roundness, yellow) and OMIM/TTD diseases (foursquare, green). Enrichment analyses of TBI and LTP were simultaneously selected. (**B**) Significantly enriched-LTP was shown. After stimulation, the opening of postsynaptic NMDAR channels lead to influx of abundant Ca^2+^, subsequently resulting in the activation of CaMKs (such as CaMKII). The activation further regulated the expression of synaptic growth-associated protein GAP-43 via stimulation of downstream signaling pathways.

Enrichment analyses were implemented to predict pathways related to the pathophysiology of TBI (Supplementary Tables 1–2). As expected, TBI (adjusted *P*-value = 1.95e–002) was significantly selected, and the corresponding pathways were targeted. Excitingly, according to KEGG enrichment analysis, one of the main form of the synaptic plasticity- LTP (adjusted *P*-value = 1.67e–003) was successfully enriched (Figure 2A–2B).

**Table 2:** Summary of the RT-PCR primers sequences

Gene	Primers	Sequences	Product length
NMDAR1	Forward	5′-GAAAGCCACATTTAGGGCTAT-3′	88 bp
	Reverse	5′-TCCACCCCCGGTGCTCGTGT-3′	
CaMK II	Forward	5′-AGACACCAAAGTGCGCAAAC-3′	133 bp
	Reverse	5′-CAGGGCCTCCGGTTCAAAGG-3′	
GAP-43	Forward	5′-GCTAGCTTCCGTGGACACAT-3′	114 bp
	Reverse	5′-ACCATCAGCAACGGGAGCAT-3′	
β-actin	Forward	5′-CATCCTGCGTCTGGACCTGG-3′	116 bp
	Reverse	5′-TAATGTCACGCACGATTTCC-3′	

### XFZYD upregulated the expression of NMADR1, CaMKII and GAP-43 after TBI

**Figure 3:**
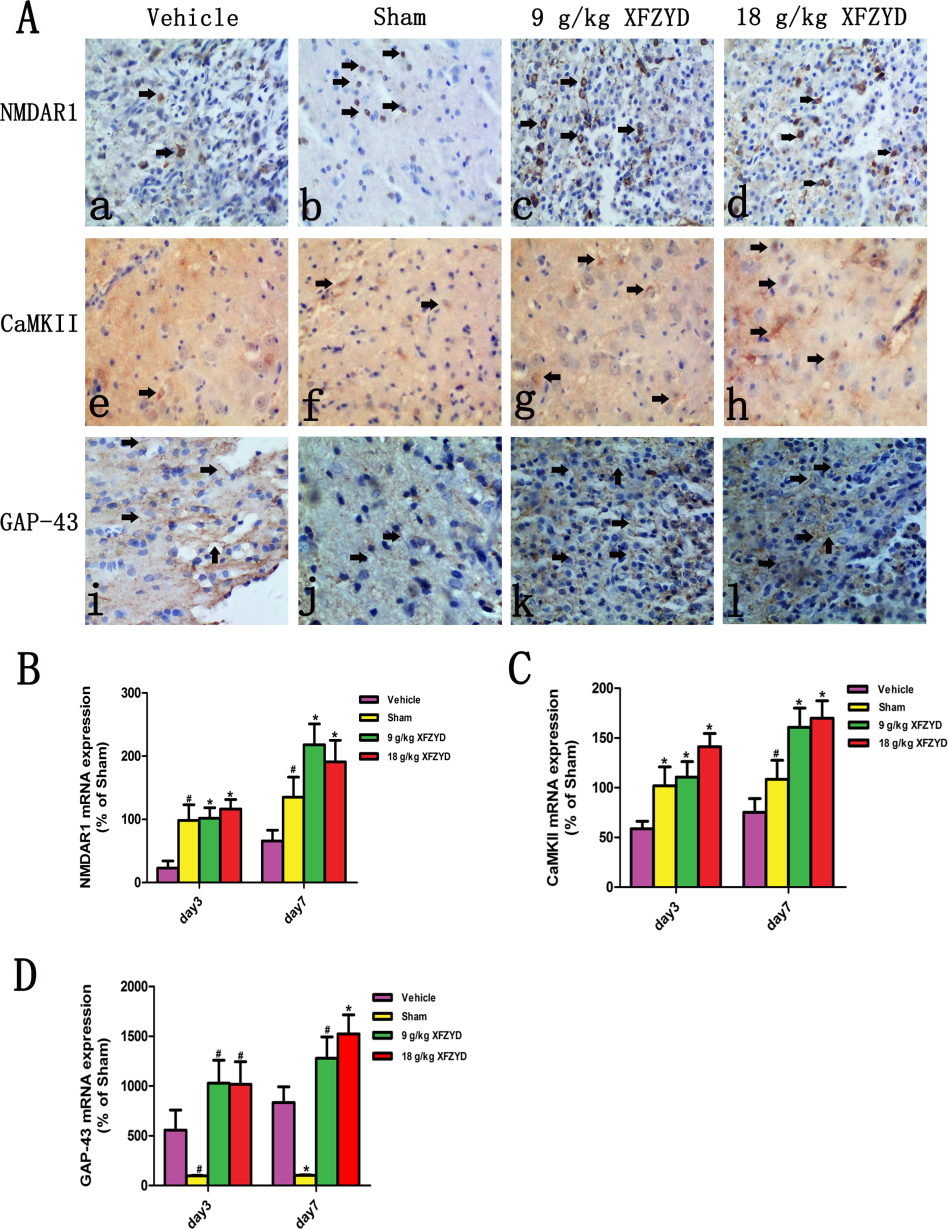
XFZYD enhanced the immunoreactivities and mRNA levels of the NMDAR1, CaMKII and GAP-43 CCI rats (**A**) Immunohistochemical analysis of NMDAR1, CaMKII and GAP-43. (b, f and j) The immunoreactivities of NMDAR1, CaMKII and GAP-43 appeared in the the Sham group. In the brains from CCI rats, rare (a) NMDAR1 and (e) CaMKII positive cells were observed. The immunoreactivities of (c and d) NMDAR1 and (g and h) CaMKII in XFZYD (9 g/kg and 18 g/kg) groups were both enhanced compared with the Vehicle group. (i) Increased immunoreactivity of GAP-43 was detected after subjected to CCI, (k and l) XFZYD (9 g/kg and 18 g/kg) significantly upregulated the expression of GAP-43 compared with the Vehicle group. RT-PCR analysis demonstrated that the downregulation of (**B**) NMDAR1 and (**C**) CaMKII mRNA, and upregulation of (**D**) GAP-43 mRNA persisted until the 7th day after TBI. XFZYD (9 g/kg and 18 g/kg) significantly elevated (B) NMDAR1, (C) CaMKII and (D) GAP-43 mRNA levels on the 3rd and 7th day compared with the Vehicle group. The values are expressed as the Mean ± SD, *n* = 5/group, *p < 0.01 and ^#^*p* < 0.05 vs. the Vehicle group.

**Figure 4:**
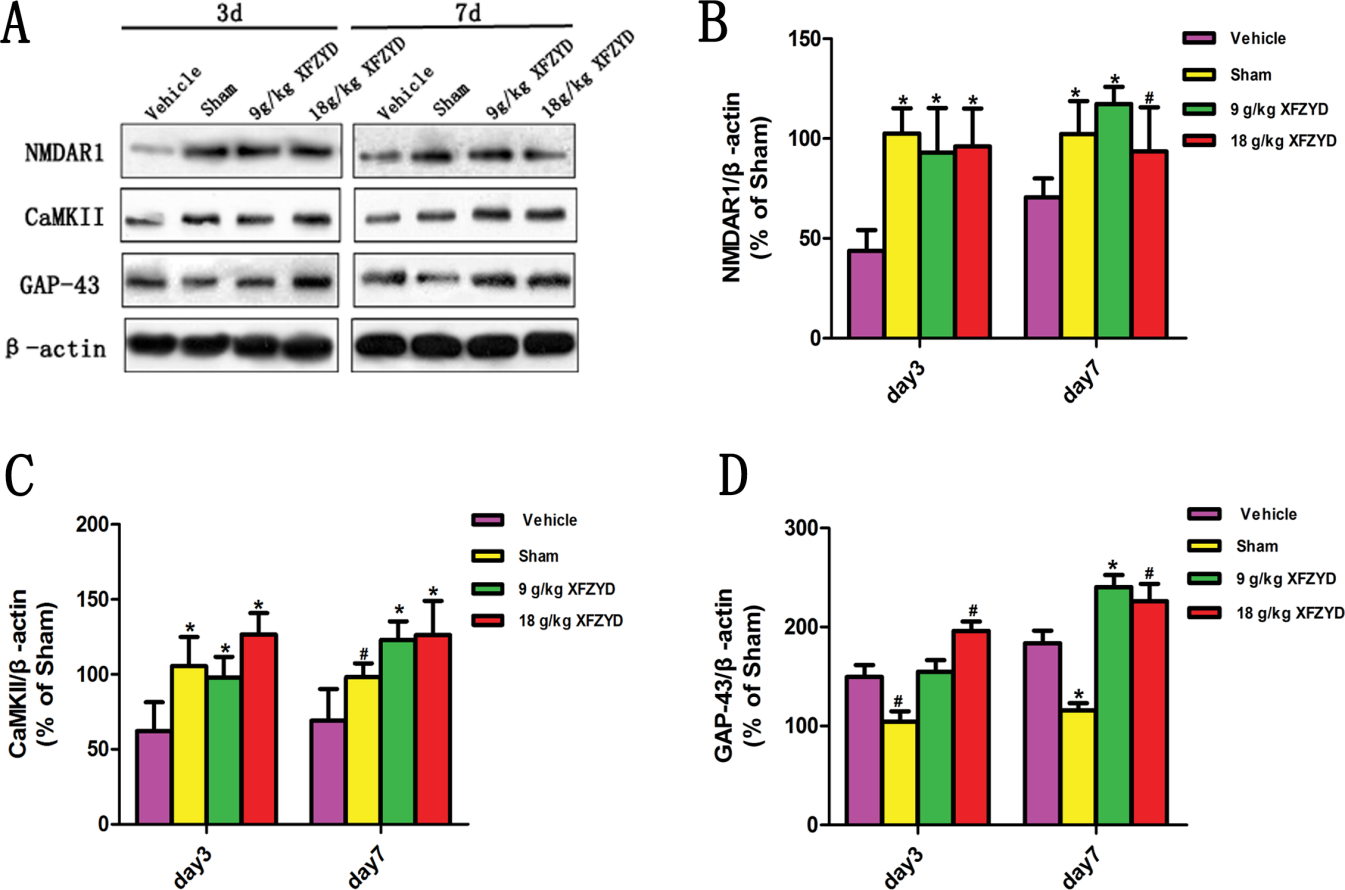
XFZYD increased NMDAR1, CaMKII and GAP-43 expressions in rat brains after TBI (**A**) Representative western blot analysis of the NMDAR1, CaMKII and GAP-43 expression in CCI rats. (**B** and **C**) XFZYD (9 g/kg and 18 g/kg) promoted the expression of NMDAR1 and CaMKII on the 3rd and 7th day compared with the Vehicle group. (**D**) 9 g/kg XFZYD elevated the levels of GAP-43 on the 7^th^ day, while 18 g/kg XFZYD on the 3rd and 7th day compared with the Vehicle group. The values are expressed as the Mean ± SD, *n* = 5/group, *p < 0.01 and ^#^*p* < 0.05 vs. the Vehicle group.

For the purpose of confirming the prediction, the results of molecular biology experiments were shown. Immunohistochemical analysis showed that reduced NMDAR1 and CaMKII, and enhanced GAP-43 expression were observed in the Vehicle group compared with the Sham group (Figure 3A). XFZYD (9 g/kg and 18 g/kg) significantly upregulated the expression of NMDAR1, CaMKII and GAP-43 positive cells (Figure 3A). The mRNA levels of NMDAR1, CaMKII and GAP-43 were measured by RT-PCR. A marked downregulation of NMDAR1 and CaMKII, as well as upregulation of GAP-43 mRNA were observed in the ipsilateral tissues on the 3rd and 7th day post TBI (Figure 3B–3D). XFZYD (9 g/kg and 18 g/kg) increased NMDAR1, CaMKII and GAP-43 mRNA levels compared to Vehicle group (Figure 3B–3D). Western blot analysis showed that XFZYD (9 g/kg and 18 g/kg) dramatically increased NMDAR1 and CaMKII levels of CCI rats on the 3rd and 7th day. 9 g/kg XFZYD elevated the levels of GAP-43 on the 7th day, while 18 g/kg XFZYD on the 3rd and 7th day compared with the Vehicle group (Figure 4A–4D).

## DISCUSSION

**Figure 5:**
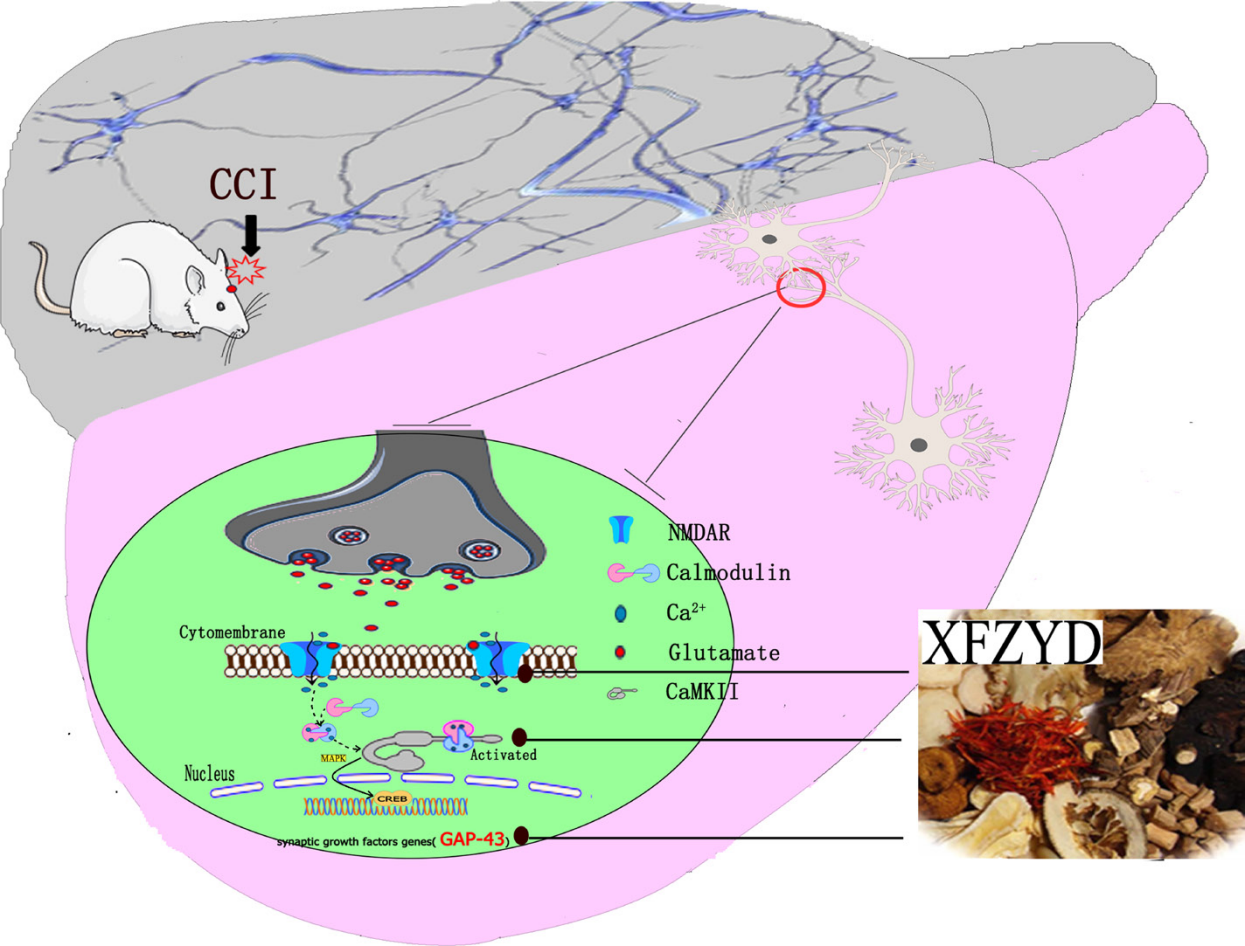
XFZYD reversed the cognitive dysfunction by improving synaptic regulation following TBI TBI reliably impaired LTP of synaptic efficacy. During the LTP process, NMDAR1 and CaMKII expressions were down-regulated. Due to the above pathophysiology, the bioinformation transmission was impaired. Our results revealed that XFZYD could markedly ameliorate cognitive function after TBI through LTP associated NMDAR1/CaMKII/GAP-43 pathway.

To the best of our knowledge, this is the first report on the synaptic regulation of XFZYD in animal model of TBI. Bioinformatics combined with molecular biology methods revealed that XFZYD improves cognitive impairment of TBI via synaptic regulation. This regulation involves the upregulation of LTP relative proteins including NMDAR1, CaMKII and GAP-43. Taken together, the results revealed that XFZYD may provide a novel therapeutic strategy for cognitive impairment after TBI (Figure 5).

Years following TBI, cognitive impairment is the most prominent complaint for the survivors [4]. According to the animal experiment, CCI rats subject to learning and memory deficits [42]. Although several promising pharmacological compounds have been identified and tested in animal studies, numbers of phase II and phase III clinical trials have failed to improve cognitive dysfunction for TBI [43–46]. Due to the complex pathomechanisms, “one-compound, one-target” based therapeutic agents fail to obtain satisfied effects in the treatment of TBI. There is an unmet need to discover novel multiple-targets based drugs to alleviate cognitive deficit post-TBI. TCM draws much attention and plays essential roles in the drug discovery and development in recent years [47, 48]. Our results indicated that the “multi-compounds, multi-targets” based XFZYD markedly ameliorated neurological and cognitive deficits after TBI. It is demonstrated that XFZYD has potentially therapeutic effects to improve cognitive function in CCI rats.

The protective function of XFZYD has been confirmed in previous study [20]. However, the sophisticated mechanisms of the above improvement treated with multi-targets based TCM make it difficult to elucidate the XFZYD neuroprotective roles in TBI [33, 49, 50]. Systematic bioinformatics method deserves to be loaded. With the help of BATMAN-TCM, an optimal systematic and comprehensive database for pharmacology analyses, we predicted the synaptic regulation of XFZYD. This regulation contains several proteins which consist of certain pathways (Figure 2A). The CIDs of the 34 identified compounds of XFZYD were submitted into BATMAN-TCM [37, 41]. Interestingly, the results showed that the main druggable target-LTP pathway was anchored by KEGG enrichment analysis. According to the previous study, memory based brain diseases are associated with the impaired LTP [51]. The current investigation showed that NMDAR1, CaMKII and GAP-43 were closely relevant to LTP, which is consistent with previous publications [26, 52].

Damage to the synaptic plasticity is one of the major deleterious mechanisms that contribute to the cognitive impairment after TBI. The NMDAR-dependent LTP is a widely used form of synaptic plasticity [53, 54]. CCI rat model reliably impairs LTP of synaptic efficacy. This deficit can be quite persistent for a long time. Following TBI, several pathological processes, including inflammatory processes, blood–brain barrier disruption, dendritic spine remodeling and synaptic proteins modification can alter the composition and function of NMDARs [55–58]. This pathophysiology impacts protein kinases-calcium/calmodulin-dependent protein kinase (CaMK) activity, and the phosphorylation of cAMP response-element binding protein (CREB). Subsequently, the above cascades lead to the change of synaptic growth-associated proteins such as GAP-43 [26, 59]. These processes may be responsible for the observed attenuation of LTP. Targeting LTP is a promising strategy to improve TBI-induced cognitive impairment.

To confirm the prediction by bioinformatics analysis, molecular biology experiments were further executed. We found a significant decrease of NMDAR1 and CaMKII in brains of CCI rats. This decline may reduce the influx and intracellular accumulation of Ca^2+^ needed to trigger LTP, resulting in the cognitive deficits after TBI. The deficiency of NMDAR stimulation was observed in the subacute and chronic stage after TBI [60]. Structurally, functional NMDAR is heteromultimers composed of two major subunits, NR1 and NR2. The NR1 subunit has all the features of NMDAR, thus constitutes the basis of NMDAR function [61]. Both pharmacological and genetic manipulations have demonstrated that CaMKII may be critical for the learning and memory [62, 63]. Decreased levels of NMDAR and CaMKII may destroy the biological information transmission in the subacute post-injured phase. The disturbed transmission is not benefit for the recovery of the brain function. Injured brain after TBI may get benefit from the stimulation of NMDAR and CaMKII in these phases. In this study, XFZYD improved the cognitive impairment through enhanced levels of NMDAR1 and CaMKII. In addition, the GAP-43 expression has profound effects on formation of new synapses and axonal regrowth. The high expression of GAP-43 is accompanied by neuronal plasticity and axon elongation after TBI [64, 65]. XFZYD dramatically increased the expression of GAP-43 compared with the Vehicle group. This change suggests that XFZYD may promote axonal regeneration following TBI. Future research should be performed to explore the absorbed bioactive components derived from XFZYD to improve cognitive impairment after TBI via LTP pathway.

In conclusion, this study showed that XFZYD could upregulate LTP associated NMDAR/CaMKII/GAP-43 pathway, subsequently ameliorate cognitive deficit after TBI. Our findings highlight a novel therapeutic strategy to improve cognitive impairment after TBI. Moreover, the method of bioinformatics predicts combined with validation of molecular biology may provide a paradigm for the mechanic exploration of TCM.

## MATERIALS AND METHODS

### Animals

**Figure 6:**
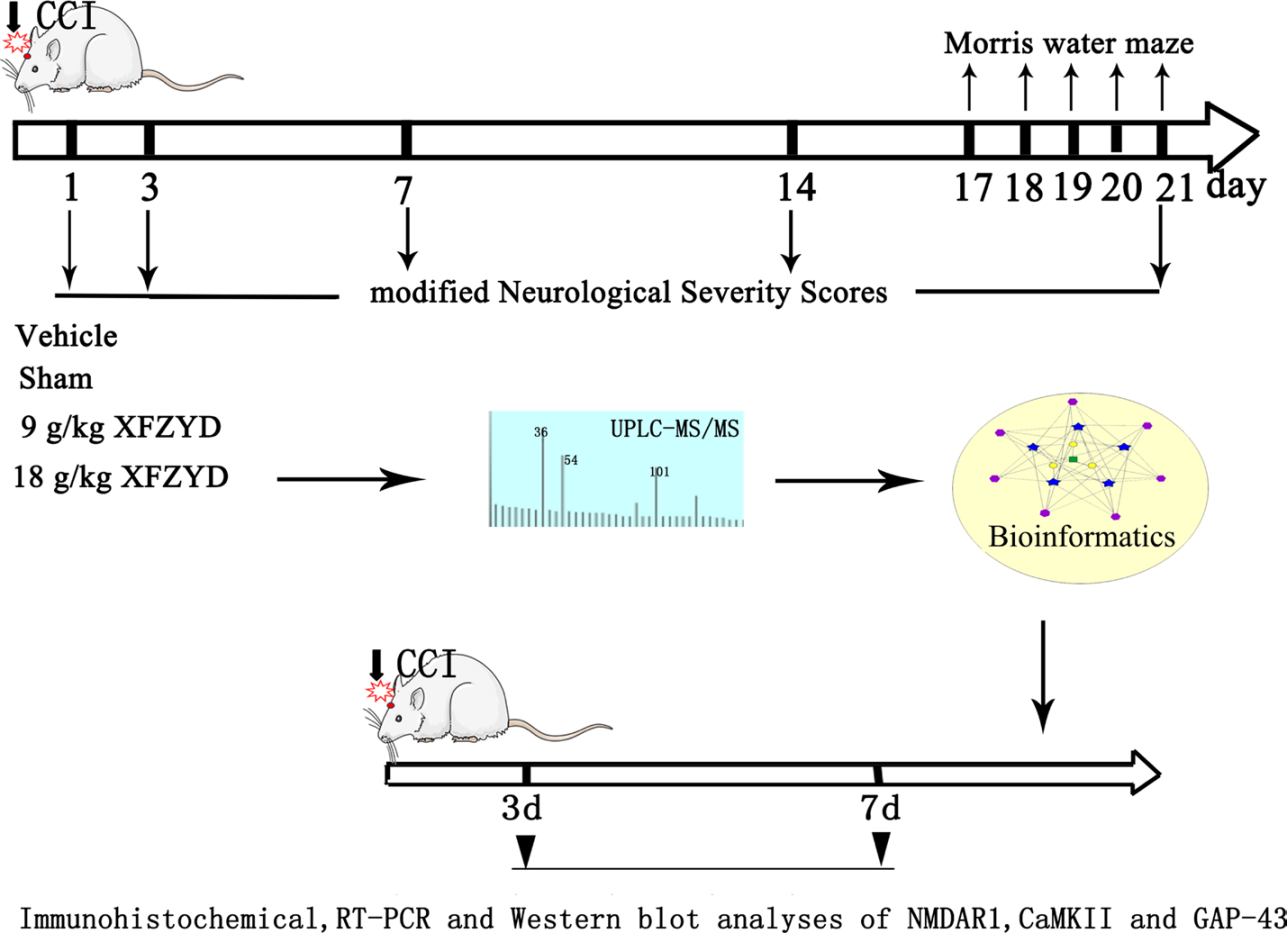
The timeline diagram depicts of the experiment Rats were randomly divided into four groups: Sham, Vehicle, 9 g/kg XFZYD and 18 g/kg XFZYD. XFZYD or distilled water was given continuously for once/day after TBI. The experiment consisted of three steps: 1. The mNSS evaluation and MWM test were performed post-TBI. 2. Bioinformatics analysis was applied to predict the effects of major ingredients from XFZYD on synaptic plasticity following TBI. 3. Molecular biology methods (including Immunohistochemical, RT-PCR and western blot analyses) were used to evaluate the expression of NMDAR1, CaMKII and GAP-43 after TBI.

Adult male Sprague-Dawley (SD) rats (200–250 g) were purchased from the Laboratory Animal Centre of Central South University (CSU). The animals were housed under controlled conditions (12-hour light/dark cycle, room temperature at 25°C and 50 ± 10% relative humidity) and allowed free access to standard rodent food and water. The experiments were performed in compliance with the guidelines for the care and use of animals established by CSU and approved by the Medical Ethics Committee of Xiangya Hospital of CSU. (The timeline diagram depicts of the experiment was shown in Figure 6).

### Experimental design and administration of drugs

Rats were randomly divided into four groups in a blinded manner: (1) Sham (*n* = 28): rats underwent the CCI procedure without cortex trauma; (2) Vehicle (*n* = 28): rats underwent CCI with cortex trauma; (3) 9 g/kg XFZYD (*n* = 28): CCI rats received XFZYD (9 g/kg) orally once a day; (4) 18 g/kg XFZYD (*n* = 28): CCI rats received XFZYD (18 g/kg) orally once a day. Sham and Vehicle groups were administered distilled water by volume equal to XFZYD groups. Two investigators who were blinded to the experimental groups recorded all of the study outcomes and performed calculation and analysis of data.

### Establishment of CCI

Rats were anesthetized with 3% pentobarbital sodium (50 mg/kg) through an intraperitoneal injection. CCI injury was induced with an electronic controlled pneumatic impact device (PSI TBI-0310 Impactor, Precision Systems & Instrumentation, Fairfax Station, VA). Animals were fixed on the stereotaxic frame with a built-in heating bed that maintained the body temperature at 37°C. A longitudinal incision along midline was created over the skull under aseptic conditions, a 5.0 mm craniotomy was generated over the left parietal cortex (center of the coordinates of the craniotomy relative to bregma: 1 mm posterior, 1 mm lateral). The impounder tip of the electronic controlled pneumatic impact device was positioned to the exposed dura, enforced the brain injury with the device parameters set as: velocity, 6 m/secs; depth, 5 mm; impact duration, 100 ms. In the Sham group, rats were underwent only the same anesthesia and craniotomy without cerebral cortex trauma.

### XFZYD preparation

Dried herbs were purchased and identified in the pharmacy of Xiangya Hospital (Hunan province, China). All herbs were processed into lyophilized powder according to the established standard procedures. Finally, each 1 g of lyophilized powder was determined to contain 5.2 g of unprocessed herbs. Lyophilized powder was diluted to the standard 1 g/ml (*w*/*v*).

### Behavioral tests

Behavioral tests were evaluated by two investigators who were blinded to the experimental groups, they scored the animals independently and finally the scores were averaged.

mNSS—Neurologic deficiency was assessed on the 1st, 3rd, 7th, 14th and 21st day after injury. The mNSS includes motor, sensory, balance, reflex tests. Test was performed based on previous study. The scores ranged from 0 to18. The higher the score, the more severe of the injury.

MWM—Spatial learning and memory deficits were conducted at the 18th∼21st day after TBI. During the first 4 days, we trained the rats to locate a hidden, submerged platform using peripheral visual information. Formal testing was performed on the 21st day after injury, time taken to reach the hidden platform with a 90 s limit was recorded. Latency to escape and swimming speed were recorded to judge the strength of the spatial learning ability. To assess spatial memory retention, a 60 s probe trial without the platform was performed on the 21st day. The number of crossing over the exact location where originally held the platform and the percentage of time spent in the target quadrant were recorded. All trials were automatically monitored and analyzed by a ANY-maze video tracking system (Stoelting Co, USA).

### Bioinformatics analysis by BATMAN-TCM combined with KEGG

To obtain the anchored pathways and proteins of XFZYD, BATMAN-TCM combined with KEGG enrichment analysis were applied. The 34 identified major compounds of XFZYD are as follows: gallic acid, l-phenylalanine, perlolyrine, chlorogenicacid, amygdalin, hydroxysafflor yellowA, albiflorin, vicenin II, verbenone, quercetin-3-glucobioside, ferulic acid, kaempferol, isoquercitrin, lonicerin, liquiritin, rutin, narirutin, naringenin, naringenin-7-O-Glucoside, naringin, hesperidin, neohesperidin, isoliquiritin, liquiritigenin, nicotiflorin, formononetin, isoliquiritigenin, sinensetin, licorice-saponin G2, senkyunolide A, 5-O-Desmethylnobiletin, glycyrrhizic acid, ligustilide and glycyrrhetinic acid (materials and results of the compounds identification are provided in Supplementary Figures 1–2). Their CIDs were simultaneously submitted to BATMAN-TCM for bioinformatics analysis.

### Brain preparation

On the 3rd and 7th day after TBI, animals were deeply anesthetized. For immunohistochemistry, animals were transcardially perfused with 0.9% ice-cold saline followed by ice-cold 4% paraformaldehyd, the removed brains were then post-fixed in 4% paraformaldehyd for 4 h. For Western bolt and RT-PCR analyses, brains were only perfused with 0.9% ice-cold saline, tissues in ipsilateral brain were immediately stored in liquid nitrogen.

### Immunohistochemical analysis

Paraffin sections were immersed in 3% hydrogen peroxide for 15 min in order to eliminate the activity of endogenous peroxidase. After nonspecific antigen blocking in 2% bovine serum albumin (BSA), sections were respectively incubated with rabbit anti-CaMK II (1:200, santa cruz), rabbit anti-NMDAR1 (1:400, Epitomics), rabbit anti-GAP-43 (1:300, Cell Signaling Technology) primary antibody at 4°C overnight, then with biotinylated-conjugated anti-rabbit secondary antibody (1:800,Proteintch) for 2 h at 37°C. Followed with avidin-biotin-peroxidase complex (ABC) (1:100, Vector) for 1 h at 37°C. Immunoreactivity was visualized with diaminobenzidine (DAB, Boster Biotech).

### Quantitative real-time RT-PCR

Total RNA was obtained from the tissues in each group using Trizol. The purity and concentration of RNA were measured with a spectrophotometer (UV-1201, Shimadzu). Reverse transcription was performed on 2 μg of total RNA with Reverse Transcription assay kit following the manufacturer's instructions (Fermentas). Amplification was performed using SYBR PCR kit (Fermentas, USA) in a Bio-Rad C × 96 Detection System (Bio-Rad, USA). The following thermocycling protocol was used: 50°C for 10 seconds,95°C for 10 minutes,40 cycles of 10 seconds at 95°C, 50 seconds at 59°C, and melting was done at 60°C. Primers for NMDAR1, GAP-43, CaMKII and β-actin were designed with Premier 5.0 software for rats. (The gene sequence of primers were shown in Table 2). Melting curves of all samples were performed as controls of specificity. The relative quantities of the candidate genes and β-actin mRNA were calculated using the comparative threshold cycle (Ct) method.

### Western blot assay

Tissues were homogenized in RIPA lysis buffer with 1 mM phenylmethyl sulfonyl fluoride (PMSF) and 1% inhibitor cocktail (Bio Basic Inc). After 30 minutes on ice, the homogenates were centrifuged for 30 min (13,000 g, 4°C). The supernatants were collected for western blot analysis. Total protein concentrations were measuered using the bicinchoninic acid (BCA, Thermo Fisher) method. Proteins were separated by SDS-PAGE and transferred onto polyvinylidene difluoride (PVDF) membranes, which were subsequently blocked in 5% skim milk for 2 h at room temperature. The membranes were incubated with the following primary antibodies: rabbit anti-CaMK II (1:500, santa cruz), rabbit anti-NMDAR1 (1:1000,Epitomics), rabbit anti-GAP-43 (1:1000, Cell Signaling Technology), mouse anti-β-actin (1:4000; abcam) for overnight, then with horseradish peroxidase-conjugated anti-rabbit secondary antibody (1:4000, Proteintch) and anti-mouse secondary antibody (1:4000,Proteintch) for 2 h at room temperature.The immunopositive bands were detected by using an enhanced chemiluminescent substrate (Thermo Fisher) and a Bio-Rad ChemiDoc XRS digital documentation system (Bio-Rad). The band density was quantified using Image J software. The amount of protein expression is presented relative to the levels of β-actin.

### Statistical analysis

All data are expressed as mean ± standard deviation (SD). A repeated-measures ANOVA (RM ANOVA) was employed for mNSS. The RM ANOVA mixed model and two-factor RM ANOVA (group × time) were used for the statistical analysis of MWM scores. The remaining biochemical data were analyzed by two-way ANOVA. The criterion for statistical significance was *p* < 0.05. Statistical analyses were conducted using the SPSS 18.0 software package or GraphPad Prism 5.0 software.

## SUPPLEMENTARY MATERIALS FIGURES AND TABLES




