# Supplementary material for: Xuefu zhuyu decoction improves cognitive impairment in experimental traumatic brain injury via synaptic regulation
Source: Oncotarget. 2017 Jun 30;8(42):72069–81. doi: 10.18632/oncotarget.18895 (PMC5641112; doi:10.18632/oncotarget.18895)
Supplement: Supplementary file 1 [file oncotarget-08-72069-s001.pdf]

Xuefu zhuyu decoction improves cognitive impairment in experimental traumatic brain injury via synaptic regulation

Supplementary Materials

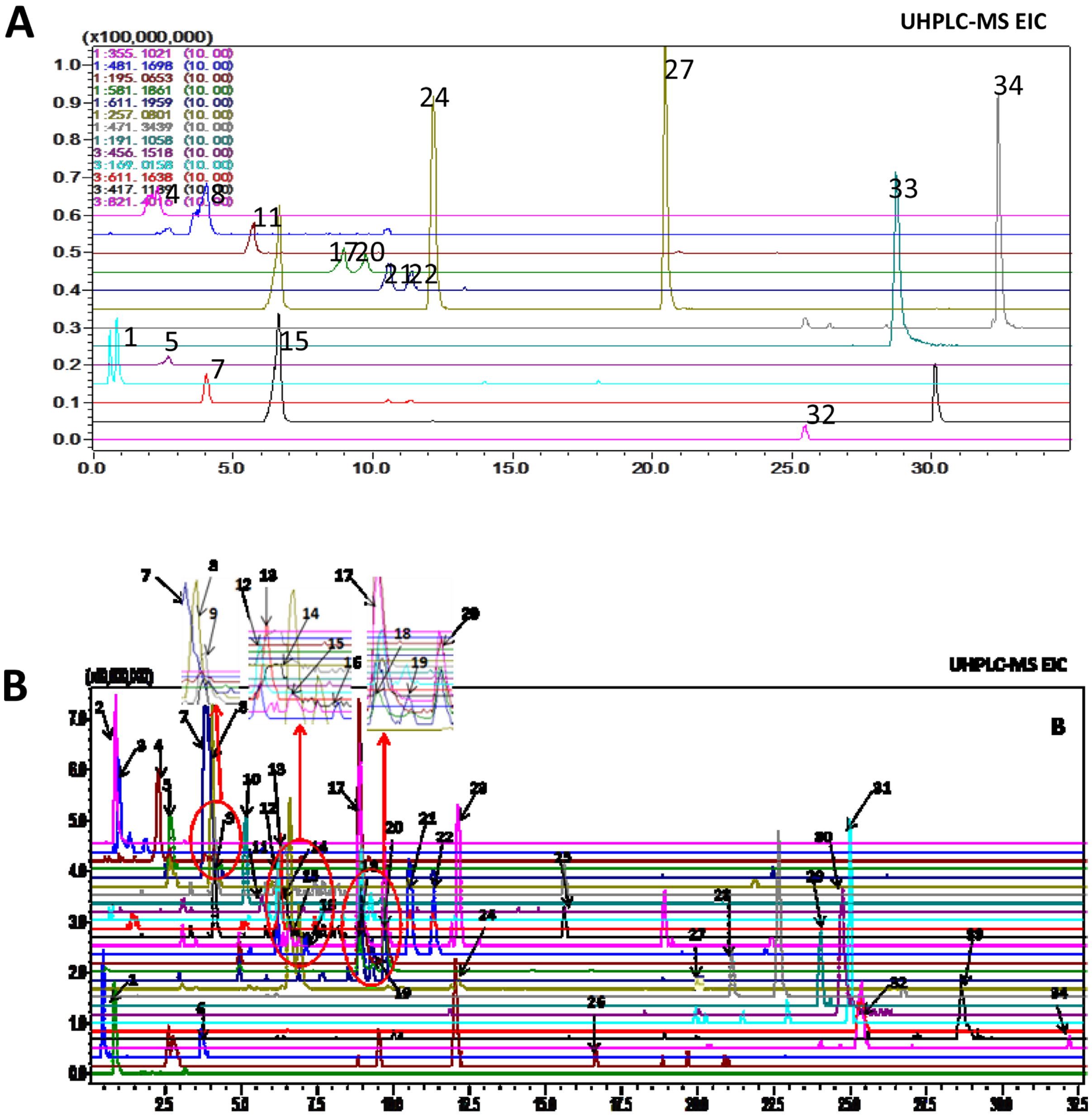

Supplementary Figure 1: UHPLC-MS EIC of reference standards mixture and XFZYD. (**A**) UHPLC-MS EIC of reference standards mixture: 1. Gallic acid; 4. Chlorogenic acid; 5. amygdalin; 7. Hydroxysafflor yellow A; 8. Albiflorin; 11. Ferulic acid; 15. liquiritin; 17. Narirutin; 20. Naringin; 21. Hesperidin; 22. Neohesperidin; 24. Liquiritigenin; 27. Isoliquiritigenin;32. Glycyrrhizic acid; 33. ligustilide ; 34. Glycyrrhetinic acid. (**B**) UHPLC-MS EIC of XFZYD: 1. Gallic acid; 2. L-phenylalanine;3. Perlolyrine; 4. Chlorogenic acid; 5. Amygdalin; 6. Verbenone; 7. Hydroxysafflor yellow A; 8. Albiflorin; 9. Vicenin II; 10. Quercetin-3-glucobioside; 11. Ferulic acid; 12. Kaempferol; 13. Isoquercitrin; 14. Lonicerin; 15. Liquiritin; 16. Rutin; 17. Narirutin; 18. Naringenin; 19. Naringenin-7-O-Glucoside; 20. Naringin; 21. Hesperidin; 22. Neohesperidin; 23. Isoliquiritin; 24. Liquiritigenin; 25. Nicotiflorin; 26. Formononetin; 27. Isoliquiritigenin; 28. Sinensetin; 29. Licorice-saponin G2; 30. Senkyunolidel; 31. 5-O-Desmethylnobiletin; 32. Glycyrrhizic acid; 33. Ligustilide; 34. Glycyrrhetinic acid.

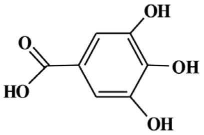

Gallic acid(1)

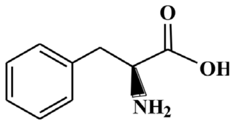

L-phenylalanine (2)

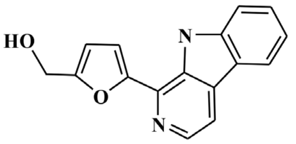

perlolyrine (3)

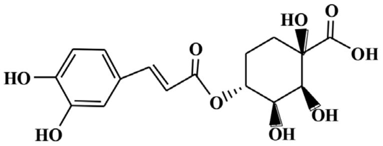

Chlorogenic acid (4)

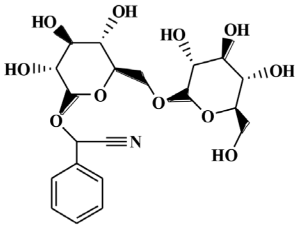

Amygdalin (5)

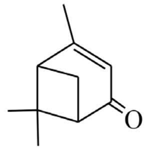

verbenone (6)

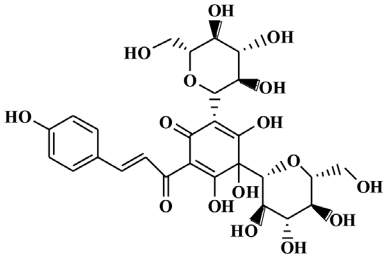

Hydroxysafflor yellow A (7)

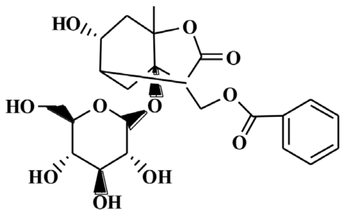

Albiflorin (8)

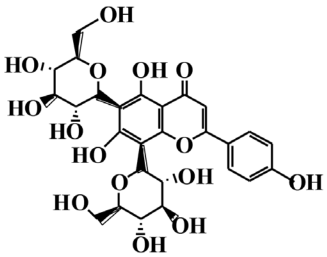

Vicenin II (9)

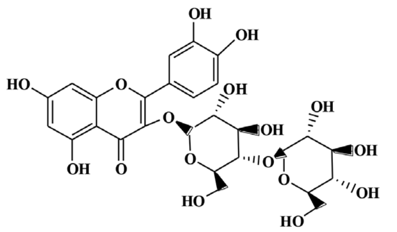

Quercetin-3-glucobioside (10)

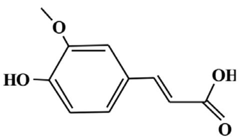

Ferulic acid (11)

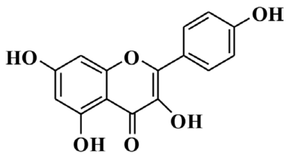

Kaempferol (12)

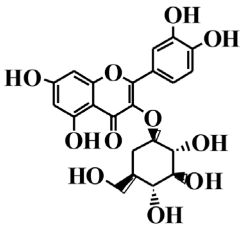

Isoquercitrin (13)

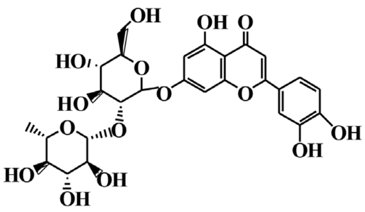

Lonicerin (14)

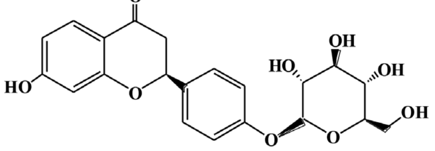

Liquiritin(15)

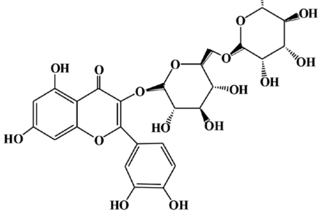

Rutin (16)

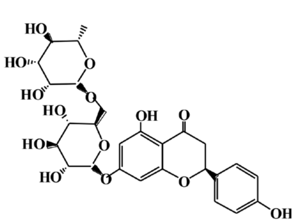

Narirutin (17)

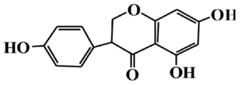

Naringenin (18)

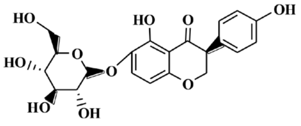

Naringenin-7-O-Glucoside (19)

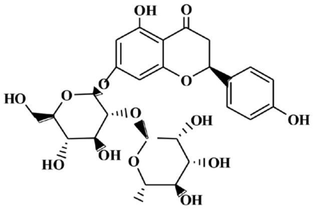

Naringin (20)

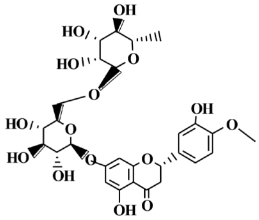

Hesperidin (21)

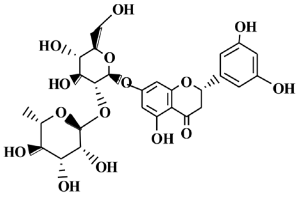

Neohesperidin (22)

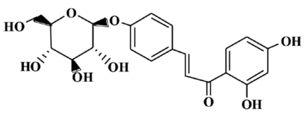

isoliquiritin (23)

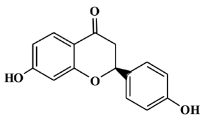

liquiritigenin (24)

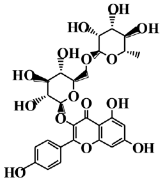

Nicotiflorin (25)

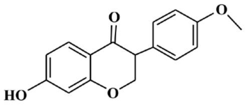

Formononetin (26)

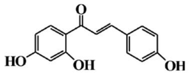

Isoliquiritigenin (27)

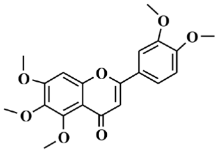

Sinensetin (28)

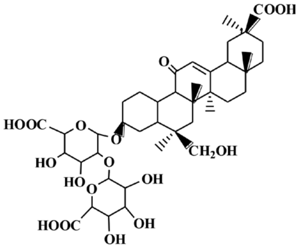

licorice-saponin G2 (29)

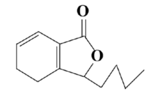

senkyunolide A (30)

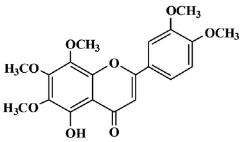

5-O-Desmethylnobiletin (31)

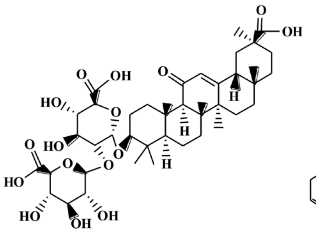

Glycyrrhizic acid (32)

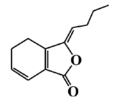

ligustilide (33)

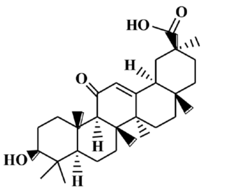

Glycyrrhetinic acid (34)

Supplementary Figure 2: The chemical structures of the 34 major compounds of XFZYD.

Supplementary Table 1: TTD disease phenotype enrichemnt analysis result of XFZYD (score >= 10).
See Supplementary_Table_1

Supplementary Table 2: KEGG pathway enrichment analysis result of XFZYD (score >= 10).
See Supplementary_Table_2
