# Supplementary material for: Xuefu zhuyu decoction improves cognitive impairment in experimental traumatic brain injury via synaptic regulation
Source: Oncotarget. 2017 Jun 30;8(42):72069–81. doi: 10.18632/oncotarget.18895 (PMC5641112; doi:10.18632/oncotarget.18895)
Supplement: Supplementary file 2 [file oncotarget-08-72069-s002.docx]

**Supplementary Table 1: TTD disease phenotype enrichemnt analysis result of XFZYD (score >= 10).** See Supplementary_Table_1

**Supplementary Table 2: KEGG pathway enrichment analysis result of XFZYD (score >= 10).** See Supplementary_Table_2
